# Clinical significance of peripheral circulating tumor cell counts in colorectal polyps and non-metastatic colorectal cancer

**DOI:** 10.1186/s12957-017-1305-2

**Published:** 2018-01-22

**Authors:** Chengguang Yang, Wenfang Zhuang, Yuemei Hu, Leiming Zhu

**Affiliations:** Department of General Surgery, Tongren Hospital, Shanghai Jiao Tong University School of Medicine, 1111 XianXia Road, Shanghai, 200336 China

**Keywords:** Circulating tumor cells, Colorectal polyps, Colorectal cancer, Pathology types

## Abstract

**Background:**

The presence of peripheral circulating tumor cells indicates the possible existence of a tumor in vivo; however, low numbers of circulating tumor cells (CTCs) can be detected in peripheral blood of healthy individuals as well as patients with benign tumors. It is not known whether peripheral CTC counts differ between patients with benign colorectal disease and those with colorectal cancer.

**Methods:**

Comparative analysis of preoperative peripheral circulating tumor cells counts was completed in patients with benign colorectal disease (colorectal polyps) and non-metastatic cancer of the colon and rectum.

**Results:**

The results of this analysis showed that patients with colorectal cancer had higher CTC counts than patients with colorectal polyps (3.47 ± 0.32/3.2 ml vs 1.49 ± 0.2/3.2 ml, *P* < 0.001). Colorectal cancer patients with tumors of the sigmoid colon displayed the highest CTC counts (4.87 ± 0.95/3.2 ml), followed by those with tumors of the rectum (3.73 ± 0.54/3.2 ml), ascending colon (3.5 ± 0.63/3.2 ml), transverse colon (2.4 ± 0.68/3.2 ml), and descending colon (2.08 ± 0.46/3.2 ml). Colorectal polyp patients with polyps in the rectum showed the highest CTC counts (2.2 ± 0.77/3.2 ml), followed by those with polyps in the ascending colon (1.82 ± 0.54/3.2 ml), sigmoid colon (1.38 ± 0.25/3.2 ml), transverse colon (0.75 ± 0.25/3.2 ml), and descending colon (0.33 ± 0.21/3.2 ml). The differences in CTC counts suggest that anatomical location of colorectal tumors may affect blood vessel metastasis. Meanwhile, patients with moderately differentiated and poorly differentiated tumors displayed higher peripheral blood CTC counts compared to those with well-differentiated tumors (*P* < 0.001). This result suggests that the type of tissue differentiation of colorectal tumors may act as another factor that affects blood vessel metastasis.

**Conclusions:**

Circulating tumor cells can be detected in the peripheral blood of colorectal cancer patients as well as patients with colorectal polyps. The differences in CTC counts suggest that anatomical location and the type of tissue differentiation of colorectal tumors may affect blood vessel metastasis.

## Background

Colorectal cancer is the most common cancer in the world [1–3], and radical surgery is the preferred treatment for patients with resectable colorectal tumors. Based on clinical pathological staging of tumors, integrated therapies can be performed subsequently, but many patients still exhibit asymptomatic tumor recurrence and metastasis within 5 years following surgery [2]. Peripheral blood circulating tumor cells (CTCs) can be detected at the time of surgery in many patients, and thus, the presence of CTCs may indicate the potential for tumor recurrence and metastasis [4–6].

The presence of peripheral CTCs suggests that a tumor may be present in vivo, and preoperative detection of peripheral blood CTCs can be used to assess blood vessel metastasis of a tumor [7–9]. When combined with postoperative clinical and pathologic staging of the tumor, surgeons can comprehensively assess the need for subsequent treatments such as chemotherapy and radiation.

However, a low number of CTCs can be detected in the peripheral blood of some healthy individuals as well as patients with benign tumors [10, 11]. Therefore, further studies are required to determine whether peripheral CTCs are differentially expressed in benign colorectal disease and non-metastatic colorectal cancer and whether detection of CTCs accurately reflects the pathological characteristics of the primary tumor in benign and malignant colorectal diseases. To address these questions, we completed a comparative analysis of peripheral CTC counts in patients with colorectal polyps and colorectal cancer.

## Methods

A total of 61 patients (36 males, 25 females) with colonic or rectal polyps, and 109 patients (63 males, 46 females) with colonic or rectal carcinoma were studied at the department of surgery of Shanghai Tongren Hospital (Shanghai, China) between December 2014 and August 2016. After acquiring patient consent and ethics committee recognition, peripheral CTC detection was performed the day before operation. Patient data and pathological tumor characteristics are listed in Table 1. Pathological consultations were completed by three experienced pathology specialists, and all tumor and polyp samples were identified as malignant epithelial tumors using Cytokeratin (Pan), Cytokeratin 7, Cytokeratin 20, and carcinoembryonic antigen antibodies. For carcinomas, the degree of tissue differentiation was classified as high, moderate, or poor differentiation, and tissue pathology was classified as papillary adenocarcinoma, tubular adenocarcinoma, papillary tubular adenocarcinoma, mucous adenocarcinoma, or signet-ring cell carcinoma. For polyps, degree of tissue differentiation was classified as low-grade intraepithelial neoplasia or high-grade intraepithelial neoplasia, and tissue pathology was classified as serrated adenoma [12], tubular adenoma, tubulovillous adenoma, mucous adenocarcinoma, or proliferative polyps.

**Table 1:** Characteristics of patients with polyps or carcinoma

Variable	Colonic and rectal polyps	Colonic and rectal carcinoma	*P* value
Patients, *n*	61	109	
Sex, *n*			
Male	36	63	*P* > 0.05
Female	25	46	
Age, years	65.83 ± 1.10 (42–81)	68.86 ± 1.12 (36–87)	*P* > 0.05
Pathologic staging		T_0-4_N_0-2_M_0_	
CTC counts(*n*/3.2 ml)	1.49 ± 0.20 (0–4)	3.47 ± 0.32 (0–15)	*P* < 0.001
Tissue differentiation, *n*	61	109	
Well differentiated		13 (11.93%)	
Moderately differentiated		76 (69.72%)	
Poorly differentiated		20 (18.35%)	
LGIEN	44 (72.13%)		
HGIEN	17 (27.87%)		
Tissue pathology, *n*	61	109	
Papillary adenocarcinoma		10 (9.17%)	
Tubular adenocarcinoma		29 (26.61%)	
Papillary tubular adenocarcinoma		47 (43.12%)	
Signet-ring cell carcinoma		1 (0.91%)	
Mucous adenocarcinoma		22 (20.19%)	
Serrated adenoma	4 (6.56%)		
Tubular adenoma	16 (26.23%)		
Tubulovillous adenoma	38 (62.3%)		
Proliferative polyps	3 (4.91%)		

Values are expressed as the mean ± standard error of the mean

*HGIEN* high-grade intraepithelial neoplasia, *LGIEN* low-grade intraepithelial neoplasia, *CTC* circulating tumor cell

### Materials and chemicals

Cytokeratin (Pan, clone lineAE1/AE3), Cytokeratin 7 (clone line OV-TL 12/30), and Cytokeratin 20 (clone line SD33) mouse anti-human cell keratin monoclonal primary antibodies, as well as carcinoembryonic antigen (clone line COL-1) mouse anti-human carcinoembryonic antigen monoclonal primary antibody, were obtained from Biocare Medical (Walnut Creek, CA, USA). Horseradish peroxidase-conjugated second antibody (goat anti-mouse) was obtained from Jackson Immuno Research Inc. (West Grove, PA, USA). LCD45-AF594 fluorescent antibody was obtained from Santa Cruz Biotechnology (Santa Cruz, CA, USA). Nuclear staining (DAPI) antibody was obtained from Beyotime Biotechnology (Shanghai, China).

### Circulating tumor cells

CTCs were enriched by negative separation. Briefly, CD45 antibody beads were used to bind white blood cells, and CTCs were enriched in precipitation. This method enriches for all CTCs, including epithelial cell adhesion molecule (EpCAM) negative, cytokine (CK) negative, and epithelial-mesenchymal transition (EMT) condition CTCs [13]. A total of 9.6 ml of blood was obtained peripherally from each patient, across three collections (3.2 ml per collection) at three time points (8 a.m., 2 p.m., and 8 p.m.), using an indwelling sheathe syringe needle on the day before operation. Peripheral blood CTC counts were measured for each 3.2 ml blood sample using fluorescence in situ hybridization, which has been routinely applied in the clinic with high stability, sensitivity, and specificity. Peripheral blood CTC counts were repeated three times, and mean values were obtained. The following criteria were used to identify CTCs: nuclear signal greater than or equal to the triploid, positive nuclear DAPI staining, and negative CD45 staining. The following criteria were used to identify white blood cells: diploid nuclear signal, positive nuclear DAPI staining, and positive CD45 staining. Staining of cells was observed and counted using a fluorescence microscope (Nikon CI-S, Tokyo, Japan).

### Pathological specimens

After surgeries and endoscopic resections, tumor tissues and polyp samples were immediately fixed in formalin for 24 hand paraffin embedded. Tissue sections were flattened on glass slides coated with poly-L-lysine. Sections were dewaxed in dimethyl benzene (30 min × 2), rehydrated in gradient alcohol (15 min intervals), washed in 1× phosphate buffer saline (PBS) (5 min × 3), and then incubated in 3% H_2_O_2_ for 10 min at room temperature to eliminate endogenous peroxidase activity. Sections were washed with 1× PBS (5 min × 3), and then incubated with 10% fetal bovine serum for 30 min. Primary antibodies (mouse anti human, 1:200) were added and incubated at 4 °C for one night. Sections were washed with 1× PBS (5 min × 3), and HRP-labeled secondary antibodies (goat anti mouse, 1:500) were added and incubated for 1 h. Sections were washed with 1× PBS (5 min × 3) and stained with diaminobenzidine for 15 min for color-developing. Sections were dyed with hematoxylin for 15 s, dehydrated in gradient alcohol at 15 min intervals, and incubated in xylene (30 min × 2). Sections were sealed with neutral resin and pictures were taken using a microscope.

### Statistical analysis

Values are expressed as the mean ± standard error of the mean. Data were analyzed with Origin 8.0 software (OriginLab Corporation). Data recordings were evaluated by two-sample *t* test. *P* < 0.05 was considered as a statistically significant result.

## Results

### Patient data and tumor characteristics

Characteristics of 61 patients with colonic or rectal polyps and 109 patients with colonic or rectal carcinoma were compared and analyzed (Table 1.). No significant difference was observed with respect to sex of the patients with colonic and rectal polyps or the patients with colonic and rectal carcinoma (*X*^2^ = 0.023, *P* > 0.05), and the ages of the patients with polyps or carcinoma were not significantly different (*t* = 1.829, *P* = 0.068). A significant difference was observed in peripheral CTC counts between patients with colonic and rectal polyps and patients with colonic and rectal carcinoma (*t* = 4.223, *P* < 0.001).

Tissue differentiation of colonic and rectal polyps was primarily classified as high-grade intraepithelial neoplasia (72.13%), whereas tissue differentiation of colonic and rectal carcinoma was mainly classified as moderately differentiated adenocarcinoma (69.72%). For tissue pathology, tubulovillous adenoma was the most common pathology in patients with colonic and rectal polyps (62.3%), whereas papillary tubular adenocarcinoma was the most common pathology in patients with colonic and rectal carcinoma (46.79%; Table 1).

### Comparison of CTC counts between colorectal polyps and colorectal carcinoma

**Fig. 1:**
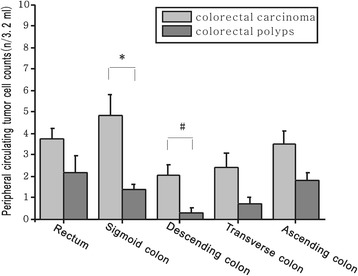
Comparison of CTC counts in colorectal polyps and colorectal carcinoma at different anatomical locations. CTC counts were higher in colorectal carcinoma than in colorectal polyps, significant differences observed in the sigmoid colon (*P < 0.001) and the descending colon (#*P <* 0.05)

According to previous reports [14] and the CTC counts in Table 1 (colorectal polyps, 1.49 ± 0.20/3.2 ml; colorectal carcinoma, 3.47 ± 0.32/3.2 ml), CTC counts were divided into two groups: > 1/3.2 ml and ≤ 1/3.2 ml. A significant difference was observed between colorectal polyps and colorectal carcinoma (*X*^2^ = 22.44, *P* < 0.001) (Table 2), with higher CTC counts observed in colorectal carcinoma, and lower CTC counts (≤ 1/3.2 ml) observed in colorectal polyps (Fig. 1.). Although there was some overlap in CTC counts between colorectal polyps and colorectal carcinoma, the difference observed was significant.

**Table 2:** CTC counts in colorectal polyps and colorectal carcinoma

	CTC counts (> 1/3.2 ml)	CTC counts (≤ 1/3.2 ml)	*X*^2^ value	*P* value
Polyps	19	42	*X*^*2*^ = 22.44	*P* < 0.001
Carcinoma	75	34		

*CTC* circulating tumor cell

### Comparison of CTC counts in colorectal carcinoma at different anatomical locations

CTC counts varied in colorectal carcinoma patients with respect to the anatomical location of the primary tumor. CTC counts were higher in colorectal carcinoma than those in colorectal polyps, with significant differences observed in CTC counts for patients with tumors of the sigmoid colon (*t* = 3.61, *P < 0.001) and the descending colon (*t* = 2.49, ^#^*P <* 0.05; Fig. 1.). In patients with tumors of the descending colon, the CTC count (0.33 ± 0.25/3.2 ml and 2.08 ± 0.46/3.2 ml) was the lowest of all colorectal tumor anatomical locations, and this trend was consistent for colorectal carcinoma and colorectal polyps (Table 3). In colorectal carcinoma, the CTC count (4.87 ± 0.95/3.2 ml) in patients with tumors of the sigmoid colon was the highest of all colorectal tumor anatomical locations. However, this was different from sigmoid colon polyps, suggesting that the anatomical location of colorectal tumors may influence the extent of blood vessel metastasis.

**Table 3:** CTC counts and tissue differentiations at different anatomical locations of colorectal polyps and carcinoma

Variable	Anatomical locations of colorectal				
	Rectum	Sigmoid	Descending	Transverse	Ascending
Average CTC counts					
Carcinoma	3.73 ± 0.54	4.87 ± 0.95	2.08 ± 0.46	2.4 ± 0.68	3.5 ± 0.63
Polyps	2.2 ± 0.77	1.38 ± 0.25	0.33 ± 0.21	0.75 ± 0.25	1.82 ± 0.38
Tissue differentiation, *n*					
Carcinoma	40	23	13	4	29
Well	5	2	3	0	3
Moderately	27	17	8	3	21
Poorly	8	4	2	1	5
Polyps	10	24	6	4	17
HGIEN	3	6	5	0	3
LGIEN	7	18	1	4	14

*Sigmoid* sigmoid colon, *Descending* descending colon, *Transverse* transverse colon, *Ascending* ascending colon, *CTC* circulating tumor cell, *HGIEN* high-grade intraepithelial neoplasia, *LGIEN* low-grade intraepithelial neoplasia

### Relationship between CTC counts and tissue differentiation in colorectal polyps and colorectal carcinoma

When CTC counts were divided into two groups (> 1/3.2 ml and ≤ 1/3.2 ml), no significant difference was observed between the two types of tissue differentiation in patients with colorectal polyps (*X*^2^ = 1.54, *P* > 0.05; Table 4). However, for carcinoma patients, those with moderately differentiated and poorly differentiated tissue types displayed significantly higher CTC counts when compared to well-differentiated tissue types (*X*^2^ = 23.39, *P <* 0.001 and *X*^2^ = 21.86, *P <* 0.001, respectively; Table 5.) Therefore, the type of tissue differentiation affected CTC counts in colorectal carcinoma, but not in colorectal polyps.

**Table 4:** CTC counts according to tissue differentiation in colorectal polyps

Polyps	CTC counts (> 1/3.2 ml)	CTC counts (≤ 1/3.2 ml)	*X*^2^ value	*P* value
HGIEN	7	10	*X*^*2*^ = 1.54	*P* > 0.05
LGIEN	11	33		

*HGIEN* high-grade intraepithelial neoplasia, *LGIEN* low-grade intraepithelial neoplasia, *CTC* circulating tumor cell

**Table 5:** CTC counts according to tissue differentiation in colorectal carcinoma

Carcinoma	CTC counts (> 1/3.2 ml)	CTC counts (≤ 1/3.2 ml)	*X*^2^ value	*P* value
Well	1	12	*X*^2^ = 23.39	*P < 0.001
Moderately	58	18	*X*^2^ = 1.79	^#^*P* > 0.05
Poorly	18	2	*X*^2^ = 21.85	^△^*P* < 0.001

*CTC* circulating tumor cell

*P represents the *P* value between well differentiated carcinoma group and moderately differentiated carcinoma group;

^#^*P* represents the *P* value between moderately differentiated carcinoma group and poorly differentiated carcinoma group;

^△^*P* represents the *P* value between well differentiated carcinoma group and poorly differentiated carcinoma group

The types of tissue differentiation of colorectal carcinoma at different anatomical locations were analyzed. In the sigmoid colon, where CTC counts (4.87 ± 0.95/3.2 ml) were the highest of all colorectal anatomical locations, moderate differentiation accounted for 73.91% (17/23), poor differentiation accounted for 17.39% (4/23), moderate and poor differentiation accounted for 91.3% (21/23), the combined percent of moderate and poor differentiation was the highest in all colorectal anatomical locations (Table 3), when compared to each other, no statistically significant difference were observed, although the ratio of moderately and poorly differentiated is highest in the sigmoid colon.

### Relationship between CTC count and tissue pathology type

Tissue pathology types of colorectal polyps included serrated adenoma, tubular adenoma, tubulovillous adenoma, and proliferative polyps. When CTC counts were divided into two groups (> 1/3.2 and ≤ 1/3.2 ml), no significant differences were observed across the tissue types (*P >* 0.05; Table 6). These results demonstrate that tissue pathology type does not affect CTC count in patients with colorectal polyps.

**Table 6:** CTC counts according to tissue pathological types in colorectal polyps

Polyps	CTC counts (> 1/3.2 ml)	CTC counts (≤ 1/3.2 ml)	*X*^2^ value	*P* value
Serrated	0	4		
Tubular	4	12	*X*^2^ = 0.71	*P > 0.05
Tubulovillous	14	24	*X*^2^ = 0.25	^#^*P* > 0.05
Proliferative	1	2	*X*^2^ = 0.17	^△^*P* > 0.05

*Serrated* serrated adenoma, *Tubular* tubular adenoma, *Tubulovillous* tubulovillous adenoma, *Proliferative* proliferative polyps, *CTC* circulating tumor cell

*P represents the *P* value between tubular adenoma polyps group and tubulovillous adenoma polyps group;

^#^*P* represents the *P* value between tubulovillous adenoma polyps group and proliferative polyps;

^△^*P* represents the *P* value between tubular adenoma polyps group and proliferative polyps

Tissue pathology types of colorectal carcinoma included papillary adenocarcinoma, tubular adenocarcinoma, papillary tubular adenocarcinoma, mucous adenocarcinoma, and signet-ring cell carcinoma. When CTC counts were divided into two groups (> 1/3.2 and ≤ 1/3.2 ml), no significant differences were observed across the tissue types (*P >* 0.05; Table 7). Therefore, tissue pathology type does not affect CTC count in patients with colorectal carcinoma.

**Table 7:** CTC counts according to tissue pathological types in colorectal carcinoma

Carcinoma	CTC counts (> 1/3.2 ml)	CTC counts (≤ 1/3.2 ml)	*X*^2^ value	*P* value
Papillary	5	5	*X*^*2*^ = 0.76	*P > 0.05
Tubular	19	10	*X*^*2*^ = 0.39	^#^*P* > 0.05
Papillary tubular	34	13	*X*^*2*^ = 0.72	^#△^*P* > 0.05
Mucous	18	4	*X*^*2*^ = 2.05	^△^*P* > 0.05
Signet-ring cell	1	0		

*CTC* circulating tumor cell

*P represents the *P* value between papillary carcinoma group and tubular carcinoma group;

^#^*P* represents the *P* value between tubular carcinoma group and papillary tubular carcinoma group

^#△^*P* represents the *P* value between papillary tubular carcinoma group and mucous carcinoma group

^△^*P* represents the *P* value between papillary carcinoma group and mucous carcinoma group

## Discussion

The importance of CTCs is becoming more apparent with recent advances in research [14–17]. Current research on CTCs involves RNA and DNA expression, cell signaling pathways, and circulating biomarkers in the blood [18–21]. Clinical detection of CTCs has been carried out in some medical institutions [22], but has been mainly applied as a tool for preliminary analysis and assessment of surgery and subsequent chemotherapy [15, 23–25]. There are limited clinical CTC studies available with large samples sizes. Although there are some studies on peripheral CTC counts in colorectal carcinoma [14, 17], few have analyzed CTC counts in patients with colorectal polyps and colorectal carcinoma, and these studies lack detailed comparative analysis in different parts of the colon and rectum. In this study, CTC counts and tumor pathological characteristics in patients with colorectal polyps or colorectal carcinoma were compared and analyzed. The results showed significantly higher peripheral CTC counts in patients with colonic and rectal carcinoma compared to patients with colonic and rectal polyps (*P <* 0.001).

Furthermore, CTC counts were noticeably different between patients with colorectal carcinoma and colorectal polyps with respect to anatomical location of the primary colorectal tumor. Specifically, CTC counts were significantly higher in colorectal carcinoma patients with tumors of the sigmoid colon (*P <* 0.001) and descending colon (*P <* 0.05) compared to colorectal polyp patients with tumors in the same location. This may result from differences in anatomy and tissue differentiation throughout the colon and rectum. Additionally, the duration of fecal retention was prolonged in the sigmoid colon, ascending colon, and rectum compared to other parts of the colon, and this was especially noticeable in patients who suffered from chronic constipation. It is possible that the prolonged duration of fecal retention resulted in stimulation of the intestinal wall, which could contribute to colonic mucosal disease [26–28]. The results also showed that tumors of the sigmoid colon were primarily moderately and poorly differentiated (combined total of 91.3% of tumors), no statistically significant difference were observed, although the ratio of moderately and poorly differentiated is highest in the sigmoid colon. Generally speaking, lower differentiated tumors had higher degrees of malignancy and more hematogenous metastasis. However, further clinical studies with large samples are required to validate this finding.

Previous studies have shown that low numbers of CTCs can be detected in the peripheral blood of some healthy individuals, as well as in patients with benign tumors [10, 11], suggesting that peripheral CTCs maybe detected in both benign and malignant colorectal disease. Indeed, CTCs were detected in patients with colorectal polyps in the present study. Furthermore, CTC counts were low or undetectable in some colorectal carcinoma patients in this study, and an overlap interval of CTC counts could be observed between patients with colorectal polyps and colorectal carcinoma. However, despite this overlap, there was a significant difference (*P <* 0.001) in CTC counts between patients with benign and malignant colorectal disease. When CTC counts were divided into two groups (> 1/3.2 and ≤ 1/3.2 ml), a significant difference was observed between colorectal polyps and carcinoma (*P <* 0.001). Specifically, higher CTC counts (> 1/3.2 ml) were observed in patients with colorectal carcinoma, whereas lower CTC counts (≤ 1/3.2 ml) were primarily observed in patients with colorectal polyps. However, when low numbers of CTCs are detectable, CTC counts cannot be used to indicate to the presence of benign or malignant colorectal disease due to the overlap in CTC counts between colorectal polyp patients and colorectal carcinoma patients. Based on the CTC counts in patients with different anatomical locations of colorectal polyps, CTC counts were highest in patients with rectal polyps (2.2 ± 0.77/3.2 ml). Therefore, the detection of peripheral CTCs could be used to indicate the presence of a malignant tumor in vivo, but only when counts are high (> 2.97/3.2 ml). Additionally, these findings confirm that peripheral CTCs can be detected not only in malignant tumors but also in benign disease, so if high peripheral CTCs detection value (> 2.97/3.2 ml) is used as a colorectal cancer screening criterion, some false positives will be eliminated as much as possible, and further research is required to determine the origin and significance of CTCs in patients with colorectal polyps.

There are very few studies assessing the application of peripheral CTC counts for determining the presence of a primary tumor and its pathological characteristics [17, 29]. One study showed that the median and overall survival of patients with peripheral blood circulating tumor cell counts ≥ 3/7.5 mL was shorter than that of patients with peripheral blood circulating tumor cell counts < 3/7.5 mL (*P* < 0.0001, [15]). This suggests that, if CTCs are uniformly distributed in the blood, a peripheral CTC count greater than 1/2.5 mL could indicate the presence of a primary tumor. Notably, this value is similar to the cutoff used in this study, where groups were divided into > 1/3.2 and ≤ 1/3.2 ml.

In order to identify the factors that influence the number of peripheral CTCs, the degree of tissue differentiation in colorectal polyps and colorectal cancer was analyzed. Colorectal polyps were classified as high-grade intraepithelial neoplasia (HGIEN) or low-grade intraepithelial neoplasia (LGIEN), where the former is considered as a precancerous lesion. Colorectal cancers were classified as highly differentiated, moderately differentiated, or poorly differentiated. In patients with colorectal polyps, the average CTC count was 1.49/3.2 ml. When CTC counts were divided into groups of > 1/3.2 and ≤ 1/3.2 ml, the degree of tissue differentiation in patients with colorectal polyps had no effect on peripheral blood CTC counts. The average CTC count in patients with colorectal cancer was 3.47/3.2 ml. When CTC counts were divided into two groups of > 1/3.2 and ≤ 1/3.2 ml, patients with moderately differentiated and poorly differentiated tumors displayed higher peripheral blood CTC counts compared to those with well-differentiated tumors. These results suggest that a lower degree of tissue differentiation in colorectal cancer tumors corresponds to higher peripheral CTC counts.

Tissue pathology in colorectal polyps and colorectal cancer was also analyzed as an additional factor that may influence peripheral CTC counts. Tissue pathologies of colorectal polyps were classified as serrated adenoma, tubular adenoma, tubulovillous adenoma, or proliferative polyps. Tissue pathologies of colorectal cancer were classified as papillary adenocarcinoma, tubular adenocarcinoma, papillary tubular adenocarcinoma, mucous adenocarcinoma, or signet-ring cell carcinoma. There was no difference between pathology types and CTC counts in colorectal polyps or colorectal carcinoma. Therefore, tissue pathology does not influence peripheral blood CTC count in colorectal disease.

## Conclusions

In summary, peripheral blood CTCs can be detected in patients with colorectal cancer as well as in patients with colorectal polyps. A significant difference in peripheral CTC counts was observed between benign and malignant disease; patients with colorectal cancer had significantly higher CTC counts than patients with colorectal polyps. High CTC counts (> 2.97/3.2 ml) correlated with the presence of a primary tumor. CTC counts varied with respect to location of the primary tumor, indicating that anatomical location and degree of tissue differentiation may affect peripheral CTC counts. Additional clinical studies with larger sample sizes are required to further validate these findings.
